# The role of public law-based litigation in tobacco companies’ strategies in high-income, FCTC ratifying countries, 2004–14

**DOI:** 10.1093/pubmed/fdv068

**Published:** 2016-10-17

**Authors:** Sarah L. Steele, Anna B. Gilmore, Martin McKee, David Stuckler

**Affiliations:** 1 Centre for Primary Care and Public Health, Barts and the London School of Medicine and Dentistry, Queen Mary University of London, London E1 2AB, UK; 2 Department for Health and UK Centre for Tobacco and Alcohol Studies, University of Bath, Bath, UK; 3 London School of Hygiene and Tropical Medicine, London, UK; 4 Department of Sociology, University of Oxford, Oxford, UK

**Keywords:** global health, governance, law, litigation, tobacco

## Abstract

**Background:**

Tobacco companies use a host of strategies to undermine public health efforts directed to reduce and eliminate smoking. The success, failure and trends in domestic litigation used by tobacco companies to undermine tobacco control are not well understood, with commentators often assuming disputes are trade related or international in nature. We analyse domestic legal disputes involving tobacco companies and public health actors in high-income countries across the last decade to ascertain the types of action and the success or failure of cases, develop effective responses.

**Methods:**

WorldLii, a publicly available online law repository, was used to identify domestic court cases involving tobacco companies from 2004 to 2014, while outcome data from LexisNexis and Westlaw databases were used to identify appeals and trace case history.

**Results:**

We identified six domestic cases in the UK, Australia and Canada, noting that the tobacco industry won only one of six cases; a win later usurped by legislative reform and a further court case. Nevertheless, we found cases involve significant resource costs for governments, often progressing across multiple jurisdictional levels.

**Discussion:**

We suggest that, in light of our results, while litigation takes up significant time and incurs legal costs for health ministries, policymakers must robustly fend off suggestions that litigation wastes taxpayers' money, pointing to the good prospects of winning such legal battles.

## Introduction

Tobacco corporations manufacture and distribute products that, when used as intended, will kill half their users.^1^ Tobacco control measures typically seek to reduce smoking initiation, especially in children, and promote cessation among users.^2^ Effective measures, set out in the Framework Convention on Tobacco Control (FCTC), include restrictions on marketing and availability, requirements to provide information, such as health warnings, and measures to increase price and regulate composition.^3^ Recent reports suggest that at least 2.3 billion people are covered by at least one tobacco control measure.^4^ While the majority of the world's governments are signatories to the FCTC, most still have far to go to implement these policies.^5^

One reason for this slow progress is because tobacco companies aggressively seek to thwart these public health efforts; tactics which the parties to the FCTC now consider its greatest barrier to implementation.^6,7^ For example, tobacco companies support misleading research^8,9^ and devote vast resources to lobbying against any legislation that might reduce their sales.^10–12^ They develop elaborate public relation campaigns, emphasizing personal choice and individual responsibility. In some cases, they even write the legislation.^13^ Where legislation is passed that challenges their interests, they seek legal remedies to prevent, or at the very least delay, the implementation or continued application of these laws.^14^

Although actions brought by Big Tobacco under investor-state dispute mechanisms and arbitration frameworks have been discussed extensively,^11,15,16^ there has been less attention to its actions in domestic courts. Here we explore the industry's use of litigation from 2004 to 2014 in high-income countries that have ratified the FCTC, including an evaluation of types of claims pursued and an account of their success or failure.

## Methods

To identify litigation strategies of major tobacco companies in domestic contexts, we searched WorldLii,^17^ a free-to-use, non-commercial, independent legal database. Unlike other databases compiled by not-for-profits that are often issue focused and therefore subject to selection bias, WorldLii integrates over 270 databases from 48 legal jurisdictions and is used by lawyers in both developed and developing settings for routine legal research. It is the largest, most jurisdictionally diverse database of domestic case law text available to legal researchers, although it does not index cases or provide details of case history. Therefore, we matched the cases WorldLii identified to LexisNexis^18^ and Westlaw,^19^ both online legal research databases available via paid subscription, which index and track the history of cases, providing any updates. In concert, these databases enabled us to evaluate the cause of action, type of law applied, and whether and how tobacco companies achieved their objectives through litigation.

Our search first applied the terms ‘tobacco’ and ‘public health’ to the WorldLii case law database, which automatically excluded statute, regulation and international law documents. This yielded 459 results, of which the case names indicated irrelevance, especially cases of individuals charged with tobacco consumption related offences. We manually excluded results if they did not (i) involve a tobacco industry party opposing a public health action; (ii) involve a decision that was purely procedural; and (iii) took place in a non-FCTC jurisdiction. We consolidated daily hearings, original hearing and appellate judgments, leaving a total of six judgments for review.

In determining the subsequent history of these judgments, we used LexisNexis and Westlaw to identify whether a case had been appealed, and if so, whether it was upheld or overturned, fully or in part. These databases also allowed us to determine whether the parties were involved in a related, but separate, case. The following information was extracted for each case: (i) the case name and reference; (ii) the jurisdiction; (iii) the cause of action; (iv) the question/s of law; (v) whether the decision was in favour of or against the tobacco industry; and (vi) the *ratio decidendi* (e.g. the legal decision). A narrative synthesis approach was used to evaluate the data, given that the qualitative nature of the findings did not permit a meta-analytic approach.

## Results

All six cases meeting the inclusion criteria relied on Public Law, considering Constitutional or Human Rights Law. These cases took place in the UK, Australia and Canada. We review each in turn.

### UK

The UK has no written constitution, so legal claims have proceeded under the legal and political bases that form the UK Constitution, including European Law. Cases have considered whether primary or secondary legislation is compatible with European law, as well as the legislation on devolution to Scotland.

In the case *British American Tobacco UK Ltd & Ors, R (on the application of) v Secretary of State for Health [2004] EWHC 2493 (Admin) UK*, the tobacco industry challenged the legality of England's Tobacco Advertising and Promotion (Point of Sale) Regulations 2004. The case took place in the High Court, involving a single bench judicial review. Tobacco parties claimed that the tobacco advertising regulations were not proportionate. European Law on proportionality requires that the measure is in pursuit of a legitimate aim; the measure be suitable to achieve the aim; the measure be necessary to achieve the aim and the measure be reasonable.^20,21^ In this case, it invoked several questions: are the Regulations disproportionate to the aim of promoting health because they allow only so limited an amount of advertisement at point of sale as to impair the ‘very essence’ of commercial free speech? Are the Regulations disproportionate because they amount to ‘too blunt an instrument’ to meet the perceived objective? And, therefore, do the Regulations infringe the laws of the European Union? Is there adequate evidence to show that the Secretary of State properly assessed for himself whether less severe regulations would be sufficient for his purposes?

The Court drew on several matters of EU Law, including decisions of national courts and the European Court of Justice. The Court found against the tobacco parties on the basis that the proportionality test was indeed satisfied. His Honour Justice McCombe accepted that the Health Minister had assessed for himself whether less severe regulations would not have satisfied the legislative purpose, and therefore, that the Minister had fulfilled the test.

Another UK case, *Sinclair Collis Ltd, R (on the application of) v Secretary of State for Health & Ors* [2011] EWCA Civ 437, involved judicial review of compatibility of primary and secondary legislation with EU Law. The Court was asked whether to impugn sections 22 and 23 of the *Health Act 2009* and the *Protection from Tobacco (Sales from Vending Machines) Regulations 2010* on the basis that they violated the principle of proportionality. On this occasion, the Court found, in a 2-1 judgment, that the public health justification was proportionate and therefore against the tobacco industry party Sinclair Collis Ltd, the largest provider of tobacco dispensing solutions in the UK. The ban was deemed justifiable by reference to the wide margin of appreciation that the principle afforded to the Government. Lady Justice Arden stated ‘… in the context of public health protection, European Union law without doubt permits a low level of intensity of review with respect to the acts of the national legislature’ [173]. However, Lord Justice Laws dissented, considering the prohibition to be disproportionate, identifying a less restrictive alternative that might have been used.

Scottish courts heard *Collis v The Lord Advocate* [2012] ScotCS CSIH_80, wherein tobacco parties claimed Section 9 of the *Tobacco and Primary Medical Services (Scotland) Act 2010* was incompatible with EU law on free movement of goods. The plaintiff, a wholly owned subsidiary of Imperial Tobacco, asked whether it was within the powers of the Scottish Parliament to prohibit the sales of tobacco from vending machines, asking whether restrictions were proportionate in terms of European Law. The Court found against the tobacco industry, finding that the measure was not disproportionate in Convention terms, as the prohibition struck a balance between the public interest in maintaining good public health and the petitioners' private economic interest in its use of vending machines and therefore was valid according to European Law.

In *Imperial Tobacco Ltd v The Lord Advocate* (Scotland) [2012] UKSC 61 UK, the tobacco industry relied on another area of UK Constitutional Law, seeking to contest the compatibility of the *Tobacco and Primary Medical Services (Scotland) Act 2010* with Scottish devolution legislation. The case involved a challenge to both sections 1 and 9 of the *Act* as being outside the legislative competence of the Scottish Parliament. The Court again found against the industry, concluding the Act was well within the competence of the Scottish Parliament.

### Australia

The tobacco industry also failed in *JT International SA v Commonwealth of Australia* [2012] HCA 43. This case considered whether Section 15 of the *Tobacco Plain Packaging Act 2011 (Cth)* resulted in an acquisition of any property of the plaintiffs otherwise than on just terms in violation of Section 51(xxxi) of the *Constitution.* The High Court found there had been no ‘acquisition’ of ‘property’, and therefore, that the Commonwealth succeeded on this point. Importantly, the plaintiffs were ordered to pay the Commonwealth's costs in this case.

### Canada

The result was similar in Canada. In *Canada (Attorney General) v. JTI-Macdonald Corp.,* [2007] 2 S.C.R. 610, 2007 SCC 30, the Court unanimously upheld new federal advertising restrictions in their entirety. However, this case stood in stark contrast to *RJR-MacDonald Inc v Attorney General of Canada* [1995] ICHRL 51, a decision over a decade earlier in which the tobacco industry also relied on human rights law to argue that the similar provisions in the *Tobacco Products Control Act, S.C. 1988* infringed the right to freedom of expression. In this earlier case, the industry party argued that the Canadian Charter of Rights and Freedoms was infringed by the legislation's banning of certain advertising and requiring unattributable health warnings, as such an infringement was not justifiable. In this case, the Courts struck down some provisions of the Act, thereby favouring the industry, finding that while the legislation validly enacted these provisions it did indeed violate the right to freedom of expression. Thus, sections 4–6 and 8–9 were unjustified and therefore inoperable.

The Canadian Parliament responded by enacting new restrictions on tobacco advertising. The new Act was similar to its predecessor, but addressed the issues raised in the earlier case. However, the tobacco industry promptly challenged it on the same grounds. In the 2007 case, the Court unanimously upheld the new federal advertising restrictions in their entirety. There were some crucial differences between these cases. In 2007, unlike 1995, the Attorney General produced voluminous evidence on the harm caused by tobacco and the role of advertising in youth initiation. Chief Justice McLachlin noted that this evidence was a significant factor in reaching her judgment, but also that this did not relieve the government of its obligation to justify the measures. She concluded, along with the majority, that the provisions were to be upheld. The result was, in effect, to overturn the tobacco industry's 1995 victory, but not to overturn the previous case.

## Discussion

### Main findings of this study

Increasing legislation to tackle the health effects of tobacco has led the industry to challenge its legality. We documented six domestic cases meeting our inclusion criteria spanning 2004–14, identifying how the dominant cause of action has been founded in Public Law, specifically Constitutional and Human Rights Law, rather than International or Private Law on trade.

While we did not focus on mapping full case histories, including procedural hearings, we note that legal disputes could result in multiple procedural and pre-trial hearings, as well as appeals across multiple jurisdictional levels in federal states like Canada and Australia. In almost all cases, the action centred on testing the compatibility of laws and regulations with documents that are either constitutions or have similar primacy over primary and secondary legislation. This is unsurprising as findings of incompatibility with primary legislation can relatively easily be addressed by amending legislation, whereas it is much more difficult to amend constitutions and the like.

### What is already known on this topic

Commentators often focus on tobacco companies' use of private and international law to prevent or delay implementation of public health reforms. Much of the existing literature documents Big Tobacco's use of Investor-state dispute settlement (ISDS) and arbitration mechanisms.^22^ This literature critiques the expansion of such dispute mechanisms on the basis that they take place in secret, rather than in open national courts, with decisions made by individuals who need not be legally trained. The relevant decision-making bodies are not constrained by precedent, and while they must not do anything that is clearly illegal, they do not reach decisions primarily on the basis of legislation or jurisprudence. This means that they are, *prima facie*, less likely to respect the safeguards for public health contained within much legislation and that outcomes are far less certain.^16^ The backgrounds of those involved may also lead them to frame disputes primarily as trade matters, with little understanding of what a health framing might look like.

The existing literature focuses on bilateral and multilateral trade agreements currently being negotiated that will increase the opportunities for ISDS and other arbitration processes.^23^ The negotiations are being conducted largely in secret, and there are serious concerns that they will increase the scope for industries producing harmful products to challenge governments seeking to reduce consumption of their products, or as the industries will frame it, appropriating their property rights, whether to trade-marked images or future revenue streams.^24^ The prospect for simultaneous domestic and international action by tobacco parties is therefore increasing.

### What this study adds

It is crucial that governments be aware that the litigation, arbitration and public relation strategies adopted by industry are intertwined. The new generation of regional trade agreements, such as Transatlantic Trade and Investment Partnership (TTIP) and the proposed Trans-Pacific Partnership (TPP), pose significant implications for tobacco litigation, and the potential to increase costs around defending public health measures generally.^25^ Alongside domestic litigation, such developments mark significant issues for government, however on the domestic front victory public health provisions usually prevail. Governments must be prepared to fend off accusations that costly litigation wastes taxpayers' money by pointing to the good prospects of success.

Our review also shows that governments must draw on expertise of those in diverse areas of law, not just trade law and arbitration, who are involved in policymaking as well as dispute processes. What is clear is that those governments regulating to reduce non-communicable diseases must expect to face simultaneous disputes in national and international fora, but that with the right expertise and effective use of the evidence, such disputes can be won.

Our results show that the tobacco industry rarely succeeds, with only one victory in 1995, undone by later law reform in Canada. Such results raise the question of why the industry engages in such litigation. One possibility is that it values the threat of litigation. Litigation is extremely complex and costly, in time and resources, for governments involved.^26^ The threat of litigation is likely to weigh heavily on governments of small, poorly resourced countries that will struggle to prepare a defence against a well-resourced transnational corporation. However, this review should encourage such governments to ignore industry threats, drawing on the persuasive precedent of these existing disputes in common law countries.

A second possibility is that litigation is a means to delay the implementation of legislation. In this respect, it would simply be a continuation of other tactics used by the industry, such as those employed to slow the passage of the European Union's 2014 Tobacco Products Directive^9^ and its 2001 predecessor.^27^ Litigation has the additional advantage of diverting the attention of the few tobacco control experts in a country. Yet there are risks in litigation for the industry. First, it is also costly for it, consuming time and money. Although the transnational tobacco companies have access to resources far in excess of health ministries, these resources are not unlimited and they must calculate how best to deploy them. Second, there is the danger that they may be forced to reveal information they would rather conceal during the process of disclosure, as happened when an advisor to the tobacco industry sued for libel in a Swiss court.^8^ Third, although now much less of a problem than in the past when the industry was still able to portray itself as being socially responsible, there is the danger of reputational risk, as happened in the notorious McLibel case where McDonalds won in the court of law but lost heavily in the court of public opinion.^28^

The use of Public Law is likely to continue, if not expand. Regulating tobacco products reveals a tension within the ever-growing field of human rights between the public interest and rights to private property and corporate freedom of expression. Such a conflict requires the courts to consider and weigh these rights; a point made most obviously in the UK cases where the test of proportionality is applied. Both at a philosophical level, where such a debate reveals the tension between human rights and neo-liberal ideology, and at a practical level, where corporations take on democratically elected governments, the issue of how far governments can or must go to protect the public interest sits central. It is therefore crucial that governments continue to defend public health action in these disputes to set precedent that is persuasive in other national level courts. This impact of fending off these disputes is no longer just nationally significant, but internationally relevant.

### Limitations of this study

The nature of legal databases precluded undertaking a fully systematic review or quantitatively assessing successes and failures on a global scale. In using WorldLii, we only search jurisdictions covered by the database, which was selected because of its ability to be used freely by those in both developed and developing settings. The database is not fully comprehensive, but as yet no legal database covers every legal jurisdiction across the world. Other databases maintained by issues-based not-for-profits like www.tobaccocontrollaws.org are cultivated and therefore subject to selection bias in routine searching and reporting.

We also limited our study to jurisdictions that have ratified the FCTC. This decision was a practical one, as not only did WorldLii cover these jurisdictions, but we also knew these countries had made formal commitments and would be the most likely sites of reform. This leaves other countries, including the USA with its many jurisdictions, as ripe sites for future research on the use of public law.

## Funding

D.S. is funded by a Wellcome Trust Investigator Award and also funded by EU HRES-313590. A.B.G.'s contribution to this work was supported by Grant Number RO1CA160695 from the US National Cancer Institute. The content is solely the responsibility of the authors and does not necessarily represent the official views of the National Cancer Institute or the National Institutes of Health.
